# Supporting Transgender and Nonbinary Residents

**DOI:** 10.1089/trgh.2018.0074

**Published:** 2019-10-09

**Authors:** Jamie D. Weinand, E. Peek Ehlinger, James F. Conniff, Ronni L. Hayon, Elizabeth Kvach

**Affiliations:** ^1^ Memorial Medical Center Family Medicine Center, Southern New Mexico Family Medicine Residency Program, Las Cruces, New Mexico.; ^2^ Alaska Family Medicine Residency, Anchorage, Alaska.; ^3^ University of Minnesota Duluth Family Medicine Residency Program, Duluth, Minnesota.; ^4^ Department of Family Medicine and Commnity Health, University of Wisconsin, Madison, Wisconsin.; ^5^ Denver Health, Denver, Colorado.; ^6^ University of Colorado Family Medicine Residency, Denver, Colorado.

**Keywords:** graduate medical education, residency, transgender, medicine, family medicine, gender identity

## Abstract

Transgender and gender nonbinary (TGNB) individuals experience challenges while applying for and completing residency, although limited research exists. An academic panel reviewed best practices for residency programs who interview and match TGNB residents. Program leadership should identify and utilize the affirming name and pronouns for all applicants, not ask gender identity during an interview unless self-disclosed, and ensure that health insurance covers transition care. Programs should proactively educate all residents, faculty, and staff on knowledge gaps regarding transgender health regardless of the presence of TGNB residents. Future studies are necessary regarding experiences of TGNB residents to investigate their specific needs.

## Background

Transgender and gender nonbinary (TGNB) individuals are people with a gender identity that differs from sex assigned at birth (SAAB).^1,2^ See Table 1 for a glossary. It is estimated that ∼0.53% of the American population identifies as transgender.^3^

**Table 1.** Glossary of Terms^a^

Term	Definition
Gender identity	A person's internal sense of their gender.
Sex	Identified by external genitalia and chromosomes.
Nontransgender person (cisgender person)	Someone whose sex assigned at birth matches their gender identity.
Gender nonbinary	Someone who does not identify as either male or female.
Gender-affirming practices	Practices that recognize a transgender or nonbinary person's gender identity as opposed to their sex assigned at birth.
Sexual orientation	Sexual attraction (distinct from gender identity).
Sexual and gender minorities	Persons whom do not identify as either heterosexual or cisgender.

^a^ Reference.^2^

Data are relatively limited regarding the experiences of TGNB resident physicians. Current data show that discrimination has been reported by residency program leadership nationwide, suggesting that TGNB likely experience marginalization. In a 2012 national survey, family medicine (FM) residency program directors (PDs) (*n*=172) stated that they would have minor 39.8% (*n*=70) or major concerns 8.5% (*n*=15) ranking TGNB applicants, and 2.8% (*n*=5) would not rank a TGNB individual despite meeting all the program's requirements.^4^

Other available data of the experiences of attending physicians and medical students, the positions before and after residency, suggest lesbian, gay, bisexual, and transgender (LGBT) individuals in these roles experience marginalization as well. Witnessed LGBT discrimination is frequently reported by medical students.^5^ A 2011 survey of medical students (*n*=5838 respondents) reported that 0.7% of students identified as TGNB^6^ and that among transgender respondents (*n*=35) the majority (60%) were not out about their identity citing “fears of discrimination” or “lack of support” as reasons.^6^ Among attending LGBT-identified physicians, discrimination is also reported; in one 2011 survey, respondents (*n*=427) reported feeling ostracized, overhearing negative comments about LGBT persons, seeing poor treatment of LGBT patients or colleagues, or experiencing workplace harassment.^7^

A panel at the Society of Teachers of Family Medicine (STFM) Conference in May 2018 convened to explore issues surrounding the residency application and training for TGNB applicants given limited research in this area. Panel members consisted of one PD, three FM faculty, and two TGNB FM residents. This article will review best practice recommendations discussed for resident physicians, summarized in Table 2.

**Table 2.** Summary of Affirming Practices for Transgender and Nonbinary Applicants and Residents

During the residency application process, program faculty, residents, and staff should identify and consistently utilize the affirming name and pronoun of all applicants regardless of gender identity.
An easily accessible nondiscrimination policy, which includes gender identity, should be provided and/or displayed on the program web site.
Residency programs should embrace all academic and clinical interests of TGNB trainees and not expect them to provide education regarding transgender health, see all TGNB patients in a practice, or focus selectively on these topics during the interview.
Asking the gender identity of an applicant during an interview is prohibited, as per the National Matching Resident Program Code of Conduct to eliminate asking questions regarding gender. If an applicant self-discloses their gender identity, interviewers should address this in an affirming way as it pertains to the application.
Programs should proactively educate all residents, faculty, and clinic staff on knowledge gaps regarding sensitivity to and knowledge of transgender health as part of a standard curriculum regardless of whether TGNB residents or patients are present in the program.
Programs should ensure that health insurance benefits cover gender transition-related care through human resources and that affirming providers are identified to provide this care.

TGNB, transgender and nonbinary.

## Recommendations

### Application process and interviews

TGNB people often use names different from their legal names, and pronouns that affirm gender identity.^8^ An important best practice is to identify and utilize these with an interview invitation and on the interview day. At the time of publication, the Electronic Residency Application Service does not collect this information in its application and would need to be requested separately by residency programs. A TGNB resident panelist shared interview experiences where name tags or forms were given with their legal (nonaffirming) name, which created significant discomfort. Programs should request affirming name and pronouns as a universal practice from applicants, reducing the number of microaggressions that TGNB applicants face through misnaming and misgendering them.

While asking about a patient's gender identity is an encouraged practice, asking if an applicant is transgender or nonbinary is prohibited.^9,10^ A question about SAAB is equivalent to asking an applicant to disclose their TGNB identity. However, if an applicant self-discloses their gender identity verbally or in their application, it is acceptable for an interviewer to discuss this as it pertains to the application.

Alternatively, avoiding the topic of gender identity when it relates to an applicant's research or professional interests would signal an unfriendly environment. Another TGNB resident noted that they had extensive research and extracurricular involvement with the transgender community and wrote a personal statement that focused on transgender health. Many open-ended questions by interviewers felt appropriate, such as, “Could you describe your research among the transgender community?” Conversely, when interviewers avoided the topic it appeared that the interviewer was either unable or unwilling to discuss this issue.

Finally, many TGNB people face distress and discrimination related to bathroom use.^11^ One TGNB resident panelist recalled a situation in which they were unable to use the bathroom throughout the interview day because no gender-neutral bathrooms were available. Program staff should inform the entire interview group where gender-neutral restrooms are located.

### Matching and supporting residents

While no data exist regarding outcomes of TGNB patients treated by TGNB physicians, the panel agreed that residents should reflect the demographics of communities they serve. In addition, while some TGNB residents may be interested in serving TGNB patients or teaching about gender identity, residency programs should not expect TGNB residents to do so. One TGNB resident panelist expressed interest in teaching these topics, whereas the other did not. All residents regardless of gender identity should learn to work with TGNB patients as part of standard curriculum and broad-spectrum training. Trainings regarding TGNB persons should be incorporated throughout curriculum, not just when the program matches a TGNB resident. Additionally, sensitivity trainings should include administrative and clinic staff, given that they interact significantly with TGNB residents and patients.

Resident panelists agreed that while some issues at their programs related to TGNB residents had been resolved, challenges remained. For example, one resident reported that their legal (nonaffirming) name was in the electronic medical record (EMR), even after this resident had asked to use an affirming name (legal middle name). Each time this resident electronically signed a note, they experienced significant distress viewing nonaffirming names. The information technology (IT) department may need to change provider information in the EMR and should be included in sensitivity trainings on these topics.

Another TGNB resident echoed that many challenges faced were logistical in nature, that is, changing names, gender markers, and photos. It is important for residency staff to be sensitive to the needs of TGNB residents and proactively address them at the beginning of residency. Accountability for these changes may fall outside the program leadership and involve departments such as IT and human resources (HR). Programs should ensure that health insurance benefits cover gender transition-related care and that affirming providers are identified through HR, as this is crucial to health and wellness.

Other issues are not easily addressed and may require an individualized plan developed in collaboration with TGNB residents, such as how a program should respond to a patient's request for female-only providers with a nonbinary resident. A TGNB panelist stated that it is an important dialogue to have with patients and providers when developing a plan. Such discussions are enabled by an open, affirming program culture and leadership regarding gender identity.

### Ranking programs

Residents reported that a transparent environment welcoming of diversity is the most important factor for feeling affirmed during interviews and training. Web sites and recruitment materials with clearly visible nondiscrimination statements including gender identity send an affirming message to TGNB applicants. In addition, one resident reported that they were more likely to rank a program higher if they could identify faculty members who provide care for TGNB patients. This signals an affirming atmosphere, and the opportunity to learn to care for TGNB patients.

TGNB residents stated their decision-making processes in ranking residency programs were complex, based on many factors outside the gender identity. Of note, resident panelists both matched into rural programs and did not feel it was necessary that the program be urban or have an LGBT track to affirm TGNB applicants.

## Discussion

Several key themes emerged during the panel discussion including: affirming practices during residency recruitment; ways of proactively supporting TGNB residents who match into a program including addressing their personal needs and relationships with patients/staff; and providing sensitivity training and didactic education to all residents and staff in a program regardless of the presence of a TGNB resident (Table 2). TGNB resident panelists reported that they had been asked about their gender identity during interviews, which should be prohibited unless applicants voluntarily disclose.

Both TGNB panelists selected programs without a dedicated TGNB health track. Literature on medical students' selection of a residency shows that decision-making when ranking programs is complex and multifactorial.^12^ Thus, all residency programs, regardless of the availability of TGNB health focus, should be prepared for diverse applicants. Guidelines from the STFM regarding culturally sensitive training for residents recommend it be offered longitudinally.^13^ This is consistent with the panel recommendation that residency programs should institute gender-affirming policies and staff sensitivity training with special attention to the recruitment process.

Increased racial and ethnic diversity among physicians may improve quality of care and access to health care for marginalized communities; this may also be true for TGNB providers.^14,15^ Reducing barriers and improving access to affirming medical training for TGNB physicians will not only help diversify the health care workforce but may also improve care for TGNB people through supportive clinical practices and advocacy for affirming nondiscriminatory policies.

The primary limitation to this article is that it represents the views of a small number of FM residents and faculty, and may not be reflective of a full range of experiences. However, given the limited research that exists regarding the experiences of TGNB residents in graduate medical education, we believe our recommendations offer reasonable preliminary guidance for residency programs and future research.

## Conclusion

TGNB residents face discrimination during the application process and have unique challenges that residencies must consider proactively. TGNB residents offer experience and knowledge to positively enhance diversity in residency training and patient care. Knowledge in this area is limited by the lack of research regarding TGNB physician experiences. Future research should focus on the experiences of TGNB physicians in residency application, training, and practice, as well as patients outcomes when treated by a diverse physician workforce.

## Abbreviations Used

EMR: electronic medical record
FM: family medicine
HR: human resources
IT: information technology
PDs: program directors
SAAB: sex assigned at birth
STFM: Society of Teachers of Family Medicine
TGNB: Transgender and gender nonbinary
